# Ginger Water Reduces Body Weight Gain and Improves Energy Expenditure in Rats

**DOI:** 10.3390/foods9010038

**Published:** 2020-01-02

**Authors:** Samy Sayed, Mohamed Ahmed, Ahmed El-Shehawi, Mohamed Alkafafy, Saqer Al-Otaibi, Hanan El-Sawy, Samy Farouk, Samir El-Shazly

**Affiliations:** 1 Department of Biotechnology, Faculty of Science, Taif University, Taif 21974, Saudi Arabia; samy_mahmoud@hotmail.com (S.S.); elshehawi@hotmail.com (A.E.-S.); dr_alkafafy@yahoo.com (M.A.); saqer-20@hotmail.com (S.A.-O.); dmrasamy@yahoo.com (S.F.); 2 Faculty of Agriculture, Cairo University, Giza 12613, Egypt; 3 Faculty of Veterinary Medicine, University of Sadat City, Sadat City 32958, Egypt; m_m_ahmed2000@yahoo.com; 4 Department of Genetics, Faculty of Agriculture, University of Alexandria, Alexandria 21526, Egypt; 5 Department of Nutrition and Clinical Nutrition, Faculty of Veterinary Medicine, Kafrelsheikh University, Kafrelsheikh 33516, Egypt; hananelsawy2011@yahoo.com; 6 Department of Biochemistry, Faculty of Veterinary Medicine, Kafrelsheikh University, Kafr Elsheikh 33511, Egypt

**Keywords:** ginger water, obesity, energy homeostasis, gene expression, rat

## Abstract

Obesity is a serious global problem that causes predisposition to numerous serious diseases. The current study aims to investigate the effect of ginger water on body weight and energy expenditure through modulation of mRNA expression of carbohydrate and lipid metabolism. A white colored liquid obtained during freeze-drying of fresh rhizomes of *Zingiber officinal* was collected and named ginger water. It was used to treat rats, then blood and tissue samples were collected from the liver and white adipose at the end of the experiment. The serum was prepared and used for biochemical assays, while tissue samples were used for RNA isolation and gene expression analysis via Reverse transcription polymerase chain reaction (RT-PCR). Results of High Performance Liquid Chromatography (HPLC) analysis of ginger water revealed the presence of chrysin and galangin at concentrations of 0.24 µg/mL and 0.53 µg/mL, respectively. Average body weight gain decreased significantly in groups that received ginger water. In addition, both total cholesterol and serum triacylglycerol were reduced in the groups that received ginger water. Furthermore, mRNA expression of Sterol regulatory element-binding protein 1 (SREBP-1c) in the liver and leptin in adipose tissues were downregulated, while those of adiponectin, hepatic carnitine palmitoyltransferase1 (CPT-1), acyl-coA oxidase (ACO), Glucose transporter 2 (GLUT-2), and pyruvate kinase (PK) were upregulated in ginger water-treated groups. These results clearly revealed the lowering body weight gain effect of ginger water, which most likely occurs at the transcriptional level of energy metabolizing proteins.

## 1. Introduction

Obesity is a complex metabolic disorder that is currently a serious global problem. Obesity has been considered a fatal lifestyle disease during the past few decades because of increasingly high-fat and caloric-rich diets as well as genetic background [1,2]. The main reason for obesity is the energy imbalance in which the energy intake is higher than the energy expenditure. The main features of obesity include excessive fat mass and raised blood lipid concentration [3]. Obesity can lead to a wide range of diseases, such as type-2 diabetes, hypertension and hyperlipidemia, and cardiovascular diseases [4]. Therefore, prevention and treatment of obesity are a great health concern worldwide.

Although physical exercise and dieting are the preferred treatments for weight loss, in practice, this method is not effectively maintained, due to busy schedules. On the other hand, surgery is not preferable due to the risk factors and high cost. Therefore, there is a shift towards an increased use of medications to reduce weight with consideration of the side effects of these medications. Currently available antiobesity drugs attack body fat in various manners. They may promote metabolism and diminish appetite or they can affect fat digestion. Consequently, both health systems and researchers targeted the advancement of effective and safe therapies for obesity [5].

Natural products have been defined with different terms in various studies; functional food [6], food supplement [7], and the recently preferred definition “nutraceuticals” [8]. Although extensive research and patenting of nutraceuticals have been going for more than a decade, they do not have precise definition [9]. Nutraceuticals, when supported by clinical trials and known mode of action, have a major role in preventing as well as supporting the drug therapy of chronic diseases. In addition, the market of nutraceuticals is growing very fast with an expected market value of $578.23 billion in 2025, although it faces challenges due to the absence of clear regulations and marking difference from food supplements. It is expected that nutraceuticals, in the future, will be approved and marketed side by side with the pharmaceuticals [10]. This indicates the need for an international consensus of regulatory framework for research, approval, safety, labelling, marketing, and use of nutraceuticals [9].

Natural plant compounds and their derivatives have been reported to treat obesity without mortality or obvious adverse impacts [11]. Plants that contain components with antiobesity activity have been used all over the world as alternative and complementary herbal therapies [12]. Herbal medicines are plant-derived raw or refined products that are used for the treatment of diseases. The antiobesity effects of many combinations of plant extracts were investigated. Most of these investigations indicated antiobesity effects, for example, decreasing body weight gain in both animals and humans. *Arachis hypogaea* decreased body weight gain, liver size, and liver triglyceride content, with an increase of fecal lipid excretion [13]. A reduction in food intake as a result of reducing appetite and an impacted hormonal status was shown with pomegranate [14].

Ginger (*Zingiber officinale* Roscoe, Zingiberaceae) is a well-known spice and flavoring material that has also been used in traditional medicine in many areas. Ethanolic extract of ginger had a reducing impact on the levels of blood glucose in rats fed on a high fat diet [15]. In addition, ginger ameliorates hyperlipidemia in diabetic rats by decreasing serum cholesterol and serum triglycerides [16,17]. Studies showed that ginger supplement improves fructose utilization-incited fatty liver [18] and adipose tissue insulin resistance in rats [19]. Ginger extract weakened the kidney injury induced by chronic fructose consumption. This was mediated by suppressing renal over-expression of proinflammatory cytokines [20]. The important active component of ginger root is the unpredictable oil and impactful phenol compounds, for example, gingerol, which is a very powerful anti-inflammatory compound [21]. Gingerol has appeared to stabilize adipocyte hormones, plasma, lipases, and lipid profiles in high fat diet induced obese rats [22].

Modern scientific research has revealed that ginger possesses various therapeutic properties, such as antioxidant effects and anti-inflammatory impacts [23]. Ginger water is obtained during the freeze-drying of ginger rhizomes as a byproduct. Its strong smell and milky color raised our attention to its potential similar biological effects to ginger extract. Most previous studies have focused on the effects of the main constituents of ginger extracts; however, there are no investigations that have specifically addressed the efficacy of the byproduct, ginger water. Therefore, this investigation aimed to study the lowering body weight gain effect of ginger water and to explore the molecular mechanisms underlying this impact through investigating the ability of ginger water to adjust mRNA expression of different genes related to carbohydrate and lipid metabolism.

## 2. Materials and Methods

### 2.1. Experimental Design

A total of fifteen ten weeks-old adult male Wistar rats were used in this study. Animals were obtained from the Experimental Animal Research Center, University of King Abdulaziz, Saudi Arabia. The animals were kept in polyethylene cages and held under laboratory conditions of 22 °C and 55% H in the animal house of Taif University, Saudi Arabia with a 12 h/12 h light/dark cycle. All animal groups were fed standard laboratory chow with free access to water. The Committee of Taif University for animal care and use has approved all procedures under the authorization number of 1-440-6145.

### 2.2. Preparation of the Ginger Water

Ginger water is not a ginger extract, but it is a byproduct obtained during lyophilization (freeze-drying) of ginger rhizomes. Fresh rhizomes of the ginger plant were washed, sliced, and freeze-dried at −60 °C. During the freeze-drying process, the condensed white colored liquid in the freeze-dryer was collected, named as ginger water, analyzed using High Performance Liquid Chromatography (HPLC), and used for the experiment.

### 2.3. HPLC Analysis of Ginger Water

The obtained ginger water was subjected to analysis using HPLC. Briefly, ginger water was filtered through syringe filters and used for HPLC analysis against nine flavonoid standards (Cyanidine chloride, Myrecitine, Quercetine, Chrysine, Malvidine chloride, Delphinidine chloride, Naringenine, Caffeic acid, and Galangin). The HPLC conditions were similar to those mentioned previously by the authors in [24]. Samples were assayed on an HPLC Hewlett-Packard Phenomenex Luna C18 column (4.6 × 250 mm, 10 µm particle size, Hewlett-Packard, Palo Alto, CA, USA). Separation was done at 12 min linear gradient from 100% of 100 mM ammonium acetate (pH 5.5) to 100% methanol. The flow rate was 1.5 mL/min and oven temperature of 35 °C with injection volume of 20 µL. Sample components were monitored at 260 nm. For calibration, standard compounds were dissolved in ethyl alcohol. Then, each peak area was converted to micrograms per mL.

### 2.4. Animal Treatment

The animals were randomly distributed into three groups of five animals each. The first group received tap water and feed ad libitum throughout the experimental period and considered as a control group. The second and third groups received ginger water at a rate of 25% and 50% (*v*/*v*) in their drinking water, respectively. Treatment proceeded for approximately a month. Body weight and the average of daily food consumption were measured weekly until the experimental period ended.

### 2.5. Sampling

By the end of treatment and before animal sacrifice, animals were fasted for 10 h. Blood samples were directly collected from retro-orbital puncture after diethyl ether anesthesia. Serum samples were arranged and stored at −80 °C until use in subsequent analysis. Then, rats were killed by decapitation. Specimens for RNA isolation were collected from liver and white adipose tissue. Samples were kept in QiaZol (Qiagen Inc., Valencia, CA, USA) and stored at −80 °C for using in gene expression analysis.

### 2.6. Biochemical Assays

Total cholesterol (TC) and serum triacylglycerol (TAG) were measured cholorametrically using commercial kits (HUMAN Gesellschaft für Biochemica und Diagnostica mbH, Wiesbaden, Germany) according to the manufacturer’s instructions.

### 2.7. Gene Expression Analysis

#### 2.7.1. RNA Extraction and cDNA Synthesis

Tissue, 100 mg, was used for isolation of total RNA using QIAzol reagent (QIAGEN Inc., Valencia, CA, USA) as explained previously [14]. RNA quality was tested by agarose gel electrophoresis. Concentration and purity of RNA were evaluated at 260 nm and by determination of OD_260/280_ ratio, respectively. For cDNA synthesis, 4 µg of RNA were used with oligo-dT primer and M-MuLV reverse transcriptase (GoScript™ Reverse Transcriptase Promega, Fitchburg, WI, USA) as described previously [25] in the PeX 0.5 Thermal Cycler (Thermo Electronic Corporation, Milford, MA, USA). The obtained cDNA was directly used for Reverse transcription polymerase chain reaction (RT-PCR) or kept at −20 °C for future use.

#### 2.7.2. Semi-Quantitative-PCR

Expression of different genes related to energy metabolism was estimated by semi-quantitative PCR using their corresponding primers (Table 1). The tested genes included pyruvate kinase (PK), sterol regulatory element-binding protein-1c (SREBP-1c), glucose transporter-2 (GLUT-2), carnitine palmitoyl transferase-1 (CPT-1), acyl-CoA oxidase (ACO), and hormone sensitive lipase (HSL). The expression of leptin as well as adiponectin was also tested. Primers were designed using the Oligo-4 computer program and nucleotide sequence published in GeneBank (Table 1). PCR was conducted in 25 µL volume using PCR GoTaq® Master Mix (Promega Co., Fitchburg, WI, USA) as detailed previously [14]. The number of cycles and annealing temperatures of primers are summarized in Table 1. Expression of GAPDH mRNA was used as a reference (Table 1). PCR products were subjected to 1% agarose electrophoresis with ethidium bromide staining. PCR product bands were photographed under UV light. The intensities of the bands were densitometerically quantified using the NIH imageJ program (https://imagej.nih.gov/ij/).

### 2.8. Statistical Analysis

Results were analyzed statistically using one-way ANOVA and Scheffe’s protected least significant difference test, by using SPSS software (SPSS version 13.0, IBM, Chicago, IL, USA) with *p* < 0.05. Results were expressed as means ± standard errors (SE).

## 3. Results

### 3.1. Chemical Composition of Ginger Water

**Figure 1:**
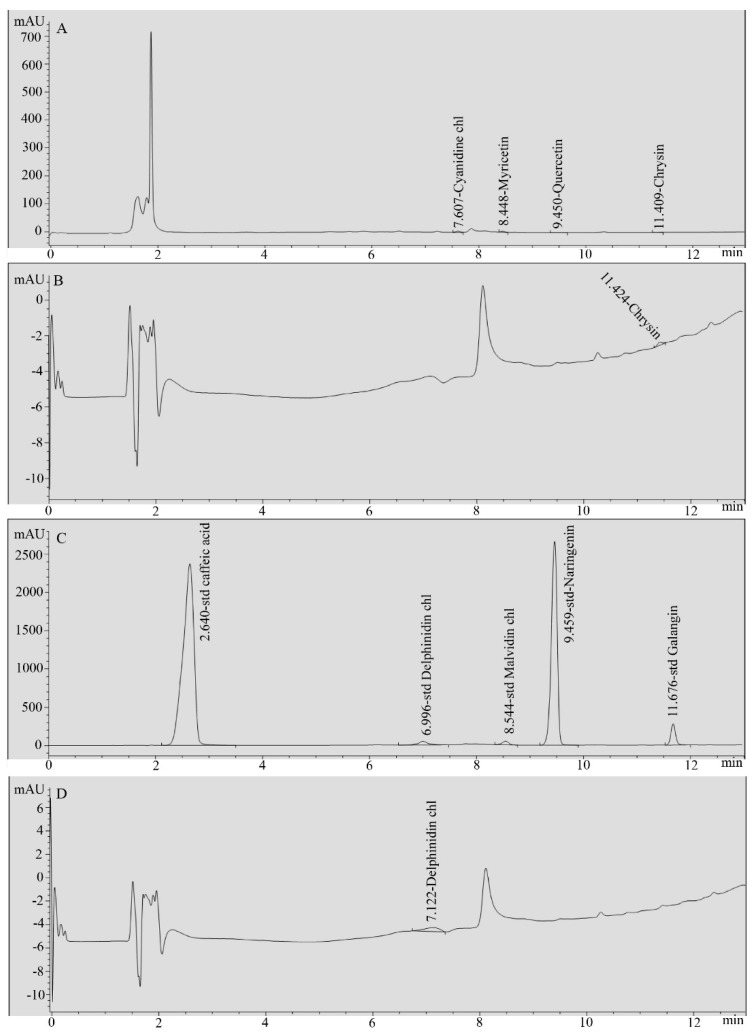
HPLC chromatograms of ginger water and reference standards. (**A**) Standard mix1, (**B**) ginger water, (**C**) standard mix2, and (**D**) ginger water.

HPLC analysis of the obtained ginger water revealed that, among the nine standards used in the HPLC analysis, only chrysin and galangin were detected in the ginger water, at concentrations of 0.24 µg/mL and 0.53 µg/mL, respectively (Figure 1).

### 3.2. Effect of Ginger Water on Food Consumption and Average Change of Body Weight

**Figure 2:**
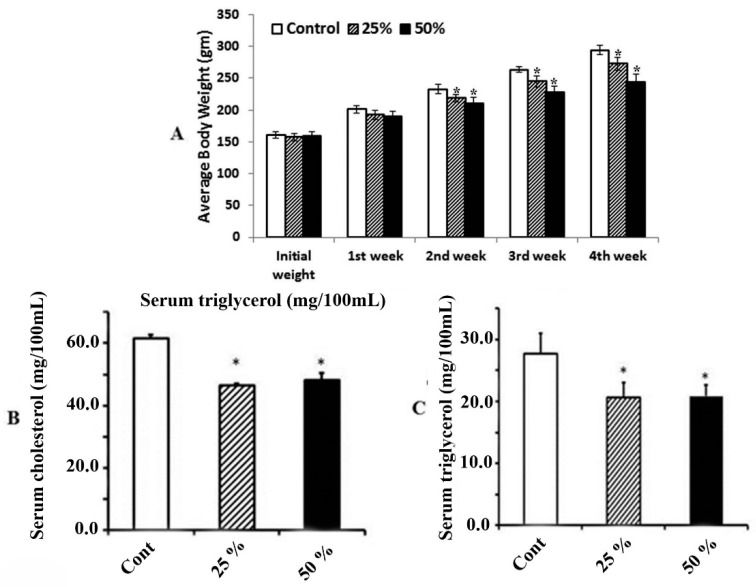
The effect of ginger water on body weight. Values are mean ± standard errors (SE) (*n* = 5). (**A**) Control, control group; 25%, 25% (*v*/*v*) ginger water-treated group; 50%, 50% (*v*/*v*) ginger water-treated group. The effect ginger water on serum level of (**B**) cholesterol and (**C**) triacylglycerol. Values are mean ± SE (*n* = 5). Cont: control, 25%:25% (*v*/*v*) ginger water-treated group, 50%:50% (*v*/*v*) ginger water-treated group. * *p* < 0.05 vs. the control.

The obtained results indicated that there was no significant decrease in neither the food consumption nor the water intake in the groups that received ginger water compared to the control. On the other hand, the weekly average body weight exhibited significant differences in the groups that received 25% and 50% ginger water compared to the control group starting from the second week (Figure 2A). The difference was indicated in the lowering body weight gain in the 25% and 50% groups compared to the control. Meanwhile, there are no significant differences among groups that received ginger water at different dose rates.

### 3.3. Effect of Ginger Water on Serum Total Cholesterol and Triacylglycerol

Administration of ginger water significantly decreased both serum total cholesterol and serum triacylglycerol compared to the control group. Meanwhile, the difference between groups that received 25% and 50% ginger water is not significant (Figure 2B,C).

### 3.4. Effect of Ginger Water Treatment on HSL and SREBP-1c mRNA Expression

**Figure 3:**
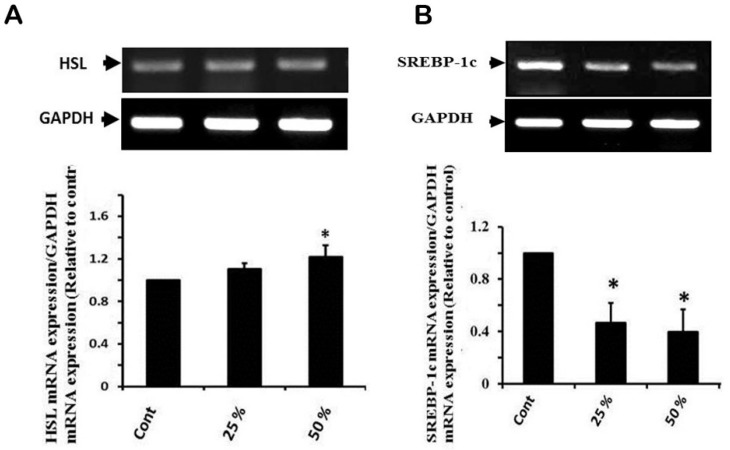
Effect of ginger water on HSL (**A**) SREBP-1c, (**B**) mRNA expressions in hepatic tissue of rats. Results of densitometric analyses and demonstrative blots of at least five independent experiments are displayed. Values are expressed as means ± SE. Cont: control, 25%:25% (*v*/*v*) ginger water-treated group, 50%:50% (*v*/*v*) ginger water-treated group. * *p* < 0.05 vs. the control.

The obtained results showed that the ginger water-receiving groups did not show significant differences with the control group in hormone sensitive lipase (HSL) mRNA expression. On the other hand, ginger water treatment at 25% and 50% induced 50% and 60% downregulation in SREBP-1c mRNA expression, respectively (Figure 3A,B).

### 3.5. Effect of Ginger Water Treatment on the Leptin, Adiponectine, and Resistin mRNA Expression in White Adipose Tissue

**Figure 4:**
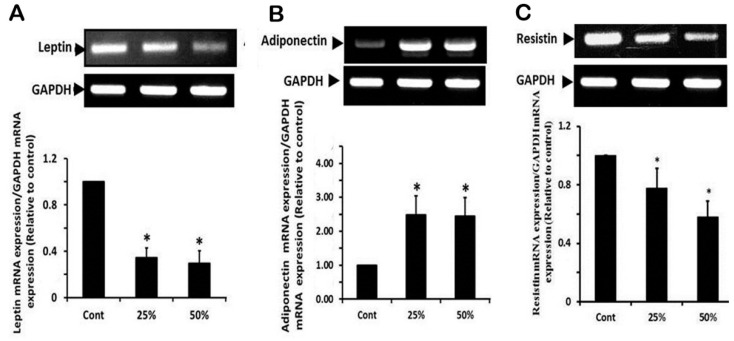
Effect of ginger water on leptin (**A**), adiponectin (**B**), and resistin (**C**), and expression of mRNA in white adipose tissue of rat. Results of densitometric analyses and demonstrative blots of at least five independent experiments are displayed. Values are expressed as means ± SE. Cont: control, 25%:25% (*v*/*v*) ginger water-treated group, 50%:50% (*v*/*v*) ginger water-treated group. * *p* < 0.05 vs. the control.

The expression of leptin mRNA was significantly downregulated (more than 3-fold) in response to receiving ginger water in both treated groups compared to the control one. Leptin mRNA expression did not show significant differences between 25% and 50% ginger water receiving groups (Figure 4A). Concerning adiponectin mRNA expression, the results showed a significant upregulation (about 2.5-fold) in groups that received ginger water compared to the control group, without significant differences between the two treated groups (Figure 4B). In the same context, ginger water treatment significantly suppressed resistin mRNA expression (Figure 4C).

### 3.6. Effect of Ginger Water Treatment on the GLUT-2 and PK mRNA Expression

**Figure 5:**
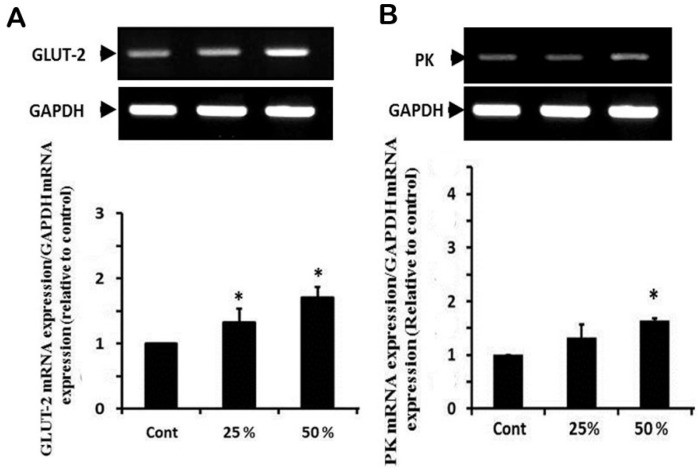
Effect of ginger water on GLUT-2 (**A**) and PK (**B**) mRNA expressions in the hepatic tissue of rats. Results of densitometric analyses and demonstrative blots of at least five independent experiments are displayed. Values are expressed as means ± SE. Cont: control, 25%:25% (*v*/*v*) ginger water-treated group, 50%:50% (*v*/*v*) ginger water-treated group. * *p* < 0.05 vs. the control.

The expression of GLUT-2 mRNA showed a significant upregulation in groups that received 25% and 50% ginger water compared to the control group (Figure 5A). In a parallel manner to GLUT-2, PK showed upregulation in groups that received ginger water that reached a significant degree in the group treated with 50% ginger water compared to the control group (Figure 5B).

### 3.7. Effect of Ginger Water Treatment on the CPT-1 and ACO mRNA Expression

**Figure 6:**
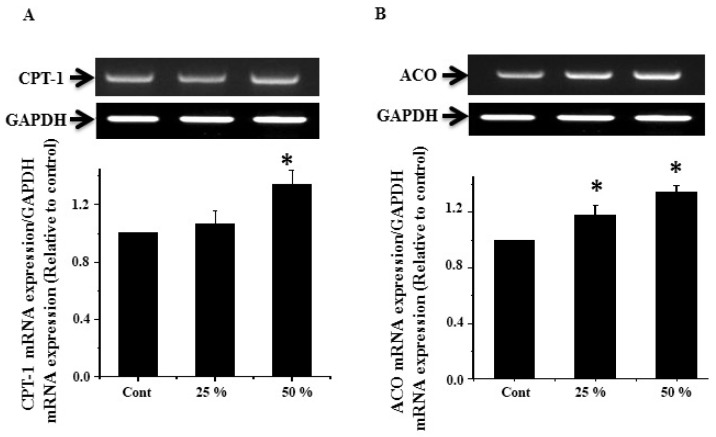
Effect of ginger water on CPT-1(**A**) and ACO (**B**) expression of mRNA in rat hepatic tissue. Results of densitometric analyses and demonstrative blots of at least five independent experiments are displayed. Values are expressed as means ± SE. Cont: control, 25%:25% (*v*/*v*) ginger water-treated group, 50%:50% (*v*/*v*) ginger water-treated group. * *p* < 0.05 vs. the control.

The expression of CPT-1 mRNA showed upregulation in the groups that received ginger water with a significant degree in the 50% group compared to the control group (Figure 6A). Similarly, ACO mRNA showed significant upregulation in groups that received 25% and 50% ginger water compared to the control group (Figure 6B).

## 4. Discussion

The use of herbal medicines has increased over the last few years for treatment of obesity. This is due to the rise in population, high cost of medicinal treatment for common disorders, side effects of different current therapeutic drugs, and the appearance of drug resistance. Ginger is considered one of the most commonly used species worldwide [26]. It belongs to the plant family that includes turmeric and cardamom. It has a strong aroma due to its high content of the pungent ketones, including gingerol, which is used in research studies as an extract [27]. Beneficial effects of ginger on obesity and its associated metabolic disorders have been shown [28,29]. It was reported that ginger extract decreases aortic atherosclerotic lesion areas, plasma cholesterol, triacylglycerol, and low-density lipoprotein [30]. In addition, ginger powder strongly decreased serum lipid levels in volunteers [31]. Moreover, ginger meal (1%) significantly lowered cholesterol levels [32]. Our obtained results showed that administration of ginger water significantly reduces the serum triacylglycerol and total cholesterol compared to those of the control group, indicating the hypolipidemic effects of ginger water. Although gingerols constitute the main portion of fresh and dry ginger, many constituents have been detected using different analytical methods [33]. In the present study, two compounds (Chrysin, Galangin) were detected in the ginger water at concentrations of 0.24 µg/mL and 0.53 µg/mL, respectively, using HPLC analysis.

Galangin is a member of the flavonol class of flavonoids and chemically known as 3,5,7-trihydroxyflavone. It is the active constituent of the rhizome of the *Alpinia galanga* plant, which belongs to the Zingiberaceae family [34]. Galangin has been proven to have various pharmacological effects [35], such as antimicrobial activity [36], anticancer [37], anti-inflammatory [38], antioxidative [39], metabolic enzyme modulation [40], and antiobesity [41] effects. Moreover, galangin was found to produce a significant decrease in serum lipids [42]. Other recent studies have revealed that galangin significantly contributed to the protection against acetaminophen-induced acute injury in liver and kidney [43]. An earlier study has shown that galangin has antioxidant activity in vitro and in vivo, free radical scavenging activity, tweaks enzymatic activity, and suppresses genotoxicity of chemicals [39].

Chrysin (C_15_H_10_O_4_) has been shown to be a very active flavonoid exerting some pharmacological properties, such as anti-inflammatory activity through blocking histamine release and expression of proinflammatory cytokines [44,45]. Antiasthmatic activity occurs via suppressing the nuclear factor-kB (NF-kB) and inducible nitric oxide synthase (iNOS) [46]. The anticancer activity of chrysin was also reported [47,48], as well antihypercholesterolemic and cardioprotective activities [49,50].

The antiobesity effect of plant preparations may act through inducing thermogenesis [51], stimulating lipolysis and decreasing lipogenesis [52], suppressing appetite [53], or decreasing lipid absorption [54]. In the current study, the administration of ginger water at a concentration of 25% and 50% showed a marked decrease in the lipogenesis process that was demonstrated by the inhibition of SEREP1c mRNA expression. The obtained data of body weights are parallel with that of leptin levels where nontreated controls showed higher body weights and leptin levels compared to the ginger water-treated groups. These results agree with that of previous studies [55].

On the contrary to leptin, adiponectin mRNA expression was higher in ginger water treated groups compared to the control group. Plasma adiponectin concentration and mRNA expression are decreased in obesity and insulin resistance [56]. Gingerol is well known to decrease serum adiponectin [57]. Therefore, this upregulated adiponectin expression could clarify the lowered blood glucose level. This might be caused by the reduced hepatic gluconeogesis and elevated insulin sensitivity [58].

Administration of ginger water apparently upregulated the hepatic mRNA expression of the lipid degradation gene, HSL, contrasted with the control. This suggested that the ginger water effects are partially caused by the downregulation of the mRNA expression of genes engaged with lipogenesis and upregulation of those concerned with lipolysis.

The lipogenic transcription factor, SREBP1c, regulates lipid metabolism via controlling the gene expression of enzymes for fatty acid synthesis, uptake, and triacylglycerol synthesis [59]. The obtained results showed a significant reduction in the mRNA expression of SREBP1c in groups that received ginger water compared to the control group. The ginger oil effectively suppressed the expression of PPARγ (Peroxisome proliferator-activated gamma), and SREBP1c [60]. Ethanolic extract of ginger reduces the levels of blood glucose in high fat diet-fed rats [15]. It has been also shown to have hypoglycemic and hypolipidemic effects in diabetic rats [16] and mice [61]. The current results showed that ginger water upregulated the expression of GLUT-2 mRNA, which plays a central role in glucose transportation from blood to liver. Moreover, Hepatic PK mRNA expression was upregulated by ginger water. PK is a key player in the glycolytic pathway. Thus, ginger water improves energy metabolism through enhancing glucose uptake via GLUT-2 mRNA expression upregulation, enhancing glucose oxidation via PK mRNA expression upregulation, and enhancing lipolysis and inhibiting lipogenesis via upregulating HSL and downregulating SREP1-c mRNA expressions, respectively. These findings could explain the obtained lipid profile in ginger water-treated groups. Moreover, ginger water could improve energy metabolism through enhancing insulin sensitivity via upregulation of adiponectin and/or downregulating both leptin and resistin expression [62,63]. These findings are in agreement with those of the previous study on the effect of vitamins A and E on lipid and carbohydrate metabolism in diet-induced obese rats [64].

The rate limiting enzyme, Acyl-CoA oxidase (ACO), catalyzes the first step in the peroxisomal β-oxidation [65]. The obtained results revealed that both 25% and 50% of ginger water resulted in upregulation of hepatic tissue ACO mRNA expression. These findings are in line with the previous work, which showed that the treatment with ginger extract led to upregulation of ACO mRNA expression, suggesting its ability to reduce liver fat accumulation through motivation of peroxisomal β-oxidation [66].

Carnitine palmitoyl transferase-I (CPT-I) is a regulatory enzyme of mitochondrial β-oxidation through controlling fatty acid transport to the mitochondrial matrix [18]. Our results revealed that ginger water administration led to upregulation of CPT-1 mRNA expression in hepatic tissue. Upregulation of cellular CPT-I expression motivated fatty acid oxidation and considerably decreased the hepatic triacylglycerol content in both high-fat diet or standard diet [67].

In conclusion, ginger water has a lowering body weight gain effect. It seems to show such activities by regulating the lipid metabolism through stimulation of lipolytic pathways and downregulation of lipogenic pathways. Additionally, ginger water may be helpful in insulin sensitization and facilitating glucose transportation to liver cells as well as improving glucose metabolism. Moreover, ginger water could have nutraceutical potential for controlling body weight, preventing obesity and obesity-associated diseases through its incorporation as food flavor, and in dietary supplements, especially for those going on a diet to lower body weight gain.

## Figures and Tables

**Table 1:** Primer sequence and PCR conditions used in this study.

Target Gene	Primer Sequence (5′–3′)	Annealing	Cycles	Product Size
GAPDH	F-AGATCCACAACGGATACATT	52 °C	25 cycles	309 bP
	R-TCCCTCAAGATTGTCAGCA			
SREP1-c	F-GGAGCCATGGATTGCACATT	58 °C	28 cycles	191 bP
	R-AGGAAGGCTTCCAGAGAGGA			
HSL	F-TGCCCAGGAGTGTGTCTGAG	61 °C	33 cycles	313 bP
	R-AGGACACCTTGGCTTGAGCG			
Leptin	F-CCTGTGGCTTTGGTCCTATCTG	59 °C	30 cycles	244 bP
	R-TATGCTTTGCTGGGGTTTTC			
Adiponectin	F-CTCCACCCAAGGAAACTTGT	52 °C	28 cycles	500 bP
	R-CTGGTCCACATTTTTTTCCT			
PK	F-ATTGCTGTGACTGGATCTGC	52 °C	28 cycles	229 bP
	R-CCCGCATGATGTTGGTATAG			
GLUT-2	F-AAGGATCAAAGCCATGTTGG	55 °C	28 cycles	330 bP
	R-GGAGACCTTCTGCTCAGTGG			
ACO	F-GCCCTCAGCTATGGTATTAC	53 °C	28 cycles	633 bP
	R-AGGAACTGCTCTCACAATGG			
CPT-1	F-TATGTGAGGATGCTGCTTCC	52 °C	28 cycles	628 bP
	R-CTCGGAGAGCTAAGCTTGCT			
